# Terahertz Spoof Surface Plasmonic Logic Gates

**DOI:** 10.1016/j.isci.2020.101685

**Published:** 2020-10-15

**Authors:** Mingrui Yuan, Qingwei Wang, Yanfeng Li, Yuehong Xu, Quan Xu, Xueqian Zhang, Xixiang Zhang, Jiaguang Han, Weili Zhang

**Affiliations:** 1 Center for Terahertz Waves and College of Precision Instrument and Optoelectronics Engineering, and Key Laboratory of Optoelectronic Information Technology (Ministry of Education), Tianjin University, Tianjin 300072, China; 2 Division of Physical Science and Engineering, King Abdullah University of Science and Technology, Thuwal 23955-6900, Saudi Arabia; 3 School of Electrical and Computer Engineering, Oklahoma State University, Stillwater, OK 74078, USA

**Keywords:** Circuit Systems, Photonics, Metamaterials

## Abstract

Logic gates are important components in integrated photonic circuitry. Here, a series of logic gates to achieve fundamental logic operations based on linear interference in spoof surface plasmon polariton waveguides are demonstrated at terahertz frequencies. A metasurface-based plasmonic source is adopted to couple free-space terahertz radiation into surface waves, followed by a funnel-shaped metasurface to efficiently couple the surface waves to the waveguides built on a domino structure. A single Mach-Zehnder waveguide interferometer can work as logic gates for four logic functions: AND, NOT, OR, and XOR. By cascading two such interferometers, NAND and NOR operations can also be achieved. Experimental investigations are supported by numerical simulations, and good agreement is obtained. The logic gates have compact sizes and high intensity contrasts for the output “1” and “0” states. More complicated functions can be envisioned and will be of great value for future terahertz integrated computing.

## Highlights

• Six major plasmonic logic gates are designed by the linear interference approach
• The logic gates are fabricated with other waveguide components on the same platform
• The logic gates are characterized using scanning near-field terahertz microscopy
• The logic gates are compact and have high-intensity contrasts for the output states

## Graphical Abstract

**Figure:**
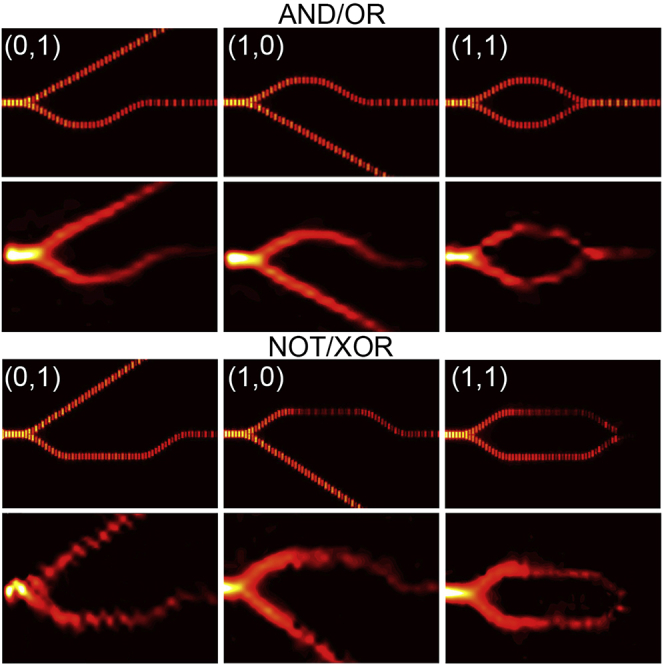

## Introduction

Terahertz (THz) technology is of great potential in developing next-generation, ultrahigh-speed communications, owing to its broad frequency band and capability in carrying ultralarge amounts of information (Nagatsuma et al., 2013; Akyildiz et al., 2014). In particular, seamless integration of THz links into existing fiber-optic infrastructures is of great importance to complement the inherent portability and flexibility advantages of wireless networks and the reliable and virtually unlimited capacity of optical transmission systems (Ummethala et al., 2019). Traditional THz devices are commonly large in volume, leading to bulky THz systems. Recent advances in metasurfaces have opened an efficient route to realizing compact devices with desired functionalities (Zheludev and Kivshar, 2012; Chen et al., 2016; Glybovski et al., 2016; Withayachumnankul and Abbott, 2009). Furthermore, the simultaneous excitation and control of THz surface plasmonic waves using metasurfaces with their wavefront control ability have greatly contributed to reducing the size of THz systems (Zhang et al., 2016; Xu et al., 2017).

THz surface plasmonic waves allow chip-scale THz manipulation, propagation, and processing simultaneously. The development of THz on-chip system based on surface plasmons is considered an important avenue for THz systems to be compact and multifunctional. This scheme is also the most promising one to achieve the simultaneous transmission of electrical and optical signals, considering the vital role THz waves might play in future communications. At the same time, THz surface plasmonic waves share similar wave properties with their optical counterparts, and thus the excitation and propagation control methods can be well transformed between the two regimes (Zhang et al., 2020). With the help of surface plasmon polaritons (SPPs), which are electromagnetic waves confined to the interface between materials with dielectric constants of opposite sign (Maier, 2007; Raether, 2013), the electromagnetic fields can be localized and manipulated at the sub-wavelength level. Traditional research on SPPs is mostly performed in the near-infrared and visible light ranges. In the THz and microwave regimes, because the dielectric constant of metals approaches that of a perfect conductor, highly confined SPPs at flat metal surfaces cannot be achieved (Jeon and Grischkowsky, 2006; Shen et al., 2008). However, Pendry et al. theoretically proved that structured metal surfaces (such as grooves and holes) can support similar SPP modes in the microwave range and referred to these modes as spoof SPPs (Pendry et al., 2004; Garcia-Vidal et al., 2005). Spoof SPPs are surface-confined electromagnetic waves supported by periodic metallic structures in the perfect-conductor limit and resemble the SPPs at a metal-dielectric interface in the optical regime in terms of field confinement and dispersion characteristics. By pattering an array of subwavelength-periodic pits, the propagation and confinement of THz electromagnetic surface modes tightly bound to flat plasmonic metamaterials can be achieved (Williams et al., 2008). A domino-like metallic structure composed of an array of box-shaped elements protruding out of a metallic surface and supporting a confined surface wave has also been proposed for this purpose (Martin-Cano et al., 2010; Brock et al., 2011). A number of designs focused on THz spoof SPP on-chip devices, including waveguiding (Maier et al., 2006; Fernández-Domínguez et al., 2009a, 2009b; Kumar et al., 2013; Zhang et al., 2017a, 2017b), S-bend waveguiding, Y-splitting, and directional coupling (Zhang et al., 2017a, 2017b), have been proposed and demonstrated. THz on-chip systems based on surface plasmons will become a promising platform to help the development and application of wireless communication in the future. However, the research in this area is still in its initial stage, and there are still a series of problems to be solved. To realize the on-chip transmission and integration of THz signals, there are a large number of complex functional devices to be accomplished, such as complex transmission devices, coupling devices, and logic gates. This is also the key to realize integrated surface plasmon systems on THz chips. Among these, logic gates are vital for future THz integrated circuits to be used in switching (Yarahmadi et al., 2015), label swapping (Ramos et al., 2005), digital processing (Zaghloul et al., 2011), computing (Tang et al., 2017), and so on.

Logic gates are the basic components of an optical signal processing system, and they are the bridges between electrical and optical calculations. Therefore, logic gates have great potential applications in the field of optical calculation and ultra-high-speed information processing. As is well known, transistor-based Boolean logic gates are the rudimentary units of electronic circuits. In photonic circuits, logic functions can be realized by linear interference effects (Caulfield, 2004; Qian and Caulfield, 2006; Zhang et al., 2007) and non-linear optical processes (Almeida et al., 2004; Xu and Lipson, 2007). For linear logic gates, the logic operation depends on the relative phase difference between two input signals (Zhang et al., 2007), where the constructive or destructive interference of the input signals determines the corresponding logic operation. The reported schemes have low field intensities and potentially high degrees of integration and also show merits of good stability and extensibility. Plasmonic microstructures can confine light into subwavelength-scale regions and exhibit strong field enhancement, which provides an approach to further scaling down photonic devices and enable direct integration with solid-state chips (Barnes et al., 2003; Zhang et al., 2012). For the high-frequency range, optical Boolean logic gates based on the interference effects of propagating plasmons on silver nanowires (Wei et al., 2011a, 2011b) and metal slot waveguides (Fu et al., 2012; Lu et al., 2013; Pan et al., 2013) have been reported. Dielectric waveguides (Birr et al., 2015), ring resonators (Godbole et al., 2016; Su and Geng, 2018; Abdulnabi and Abbas, 2019), and photonic crystals (Pirzadi et al., 2016; Rani et al., 2017) have also been proposed. However, to date, logic gates based on spoof SPPs in the THz regime have rarely been reported owing to the lack of an effective and convenient near-field characterization method. Although previous studies provide ingenious solutions to the Boolean logic operation, most of them focus on SPP waveguides for optics. It is still a great challenge to achieve compact and broadband logic gates with high performance in the THz frequency range.

In this work, we report on the design, simulation, and experimental characterization of a whole set of fundamental logic gates formed by THz spoof SPP waveguide structures. These waveguides can support transverse-magnetic (TM)-like SPP modes, and these SPP modes propagate in the direction parallel to the gold film and are suitable for on-chip integration applications. A single Mach-Zehnder interferometer can work as AND, OR, NOT, and XOR logic gates. NAND and NOR operations can be achieved by cascading two Mach-Zehnder interferometers with one arm used for the control beam. The working principle is discussed in detail and verified by experiment. These compact logic devices are stable and robust, meeting the requirements for future on-chip integration applications.

## Results

### Overall Device Design and Analysis

**Figure 1:**
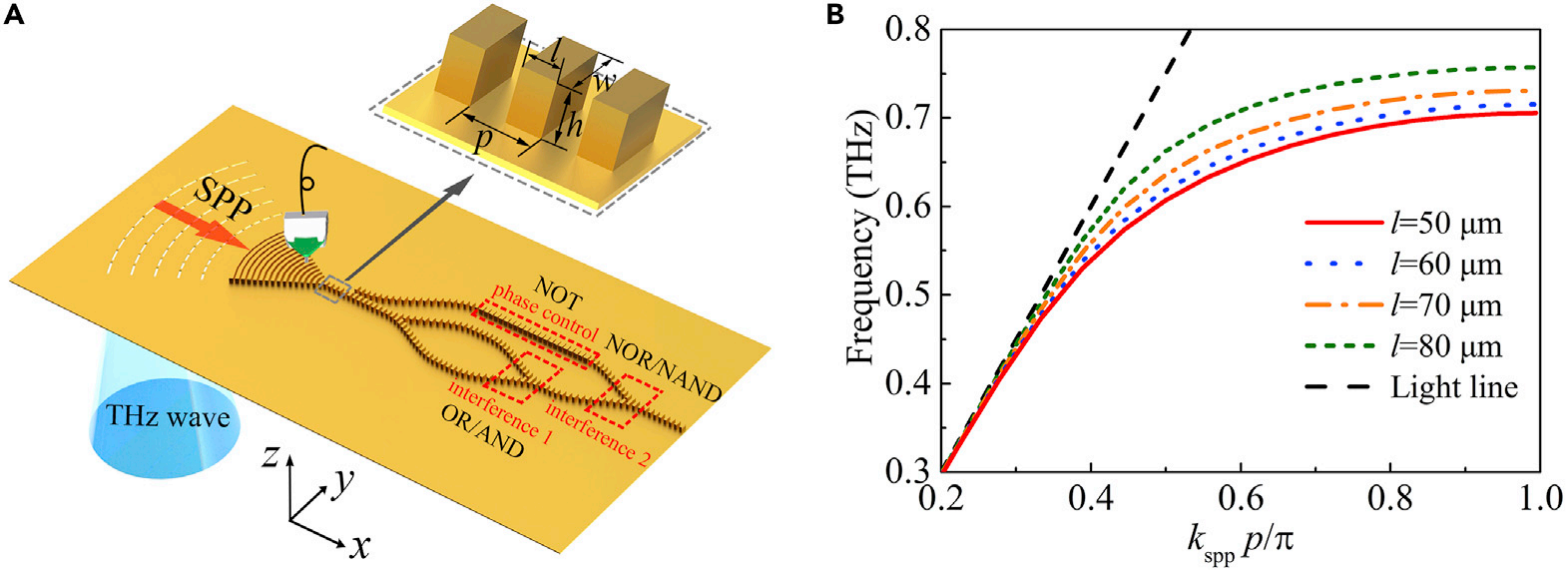
Structure and Dispersion Relation (A) Illustration of the experimental setup. The inset shows a schematic of the waveguide structure with the following geometrical parameters: *w* = 120 μm, length *l* = 50 μm, height *h* = 80 μm, and period *p* = 100 μm. (B) Dispersion relation of SPP modes for one row of metal pillars with different lengths of 50, 60, 70, and 80 μm. See also Figures S1–S3.

A schematic of the spoof SPP waveguide-based logic devices is shown in Figure 1A. Owing to the difficulty in obtaining two or more THz inputs with the same phase and amplitude and further exciting SPP waves in several regions simultaneously, the logic devices are composed of two sections: a metasurface-based excitation and focusing section, and a logic operation region based on linear interference in the spoof SPP waveguides. The waveguiding sections consist of a periodic arrangement of metallic pillars with a width *w* = 120 μm, length *l* = 50 μm, and height *h* = 80 μm arranged on the top of a metallic surface, also known as a domino structure (Martin-Cano et al., 2010; Zhang et al., 2017a, 2017b). The inset of Figure 1A shows a schematic of the metallic pillars, which are made of the same material.

Owing to the lack of direct SPP sources in the THz frequency range, free-space THz waves need be coupled into SPPs to fulfill the requirement of momentum match. The recently developed metasurface-based schemes for the excitation and manipulation of THz SPPs provide a new platform for THz SPPs (Xu et al., 2016, 2017; Zhang et al., 2015). In particular, subwavelength metallic slits are among the most commonly used unit elements in designing such plasmonic metasurfaces. Here, an arc-shaped curved slit array on the thin metal is adopted to couple free-space THz radiation into surface waves, where curved slits with a width of 40 μm are arranged with a period of 400 μm along the radial direction. The innermost and outermost radii of the annular sector region are 2,220 and 3,820 μm, respectively, and the central angle is 60°. As the source, a linearly polarized THz wave with its polarization direction parallel to the propagation direction of the waveguide is irradiated vertically from the bottom side of the sample to the slit array to eliminate the interference with the free-space THz wave. An eigen-analysis by the eigen-mode solver is provided in Figure S1 for the characterization of the excitation properties of the metallic slit resonator. The capability of metasurfaces in phase control for free-space waves can serve as a bridge to achieve momentum match between free-space THz waves and SPPs. Besides the structures used for the SPP source, metasurfaces can also be used to control the THz SPPs. To efficiently couple the SPPs to the waveguides, an arc-shaped metasurface composed of the same metallic columns as those in the waveguiding sections are employed in this work (Yuan et al., 2019, 2020). Since the effective index of the SPP mode is rather insensitive to the lateral width of the pillar (Martin-Cano et al., 2010), the compact arc-shaped metasurface is able to laterally compress the mode size down and couple the SPPs efficiently into the waveguiding section, as shown in Figures S2 and S3.

The dispersion relation for the fundamental mode of the waveguide is calculated by the commercial software CST Microwave Studio, and the results are depicted in Figure 1B. In all simulations, the metal is simplified as a perfect electric conductor, which is valid for metals in the microwave and THz regions (Martin-Cano et al., 2010; Zhang et al., 2017a, 2017b). In Figure 1B, the length of the metallic pillar *l* is varied from 50 to 80 μm to investigate its effect on the surface wave propagation. Note that as the frequency increases, the dispersion curves of the metallic pillars with different *l* are gradually separated. The wave vector of the SPP for the unit with a larger length is smaller than that for the unit with a smaller length, which means that we can control the THz surface waves simply by changing the unit length in the waveguide.

### Logic Gates with Two Input Ports

**Figure 2:**
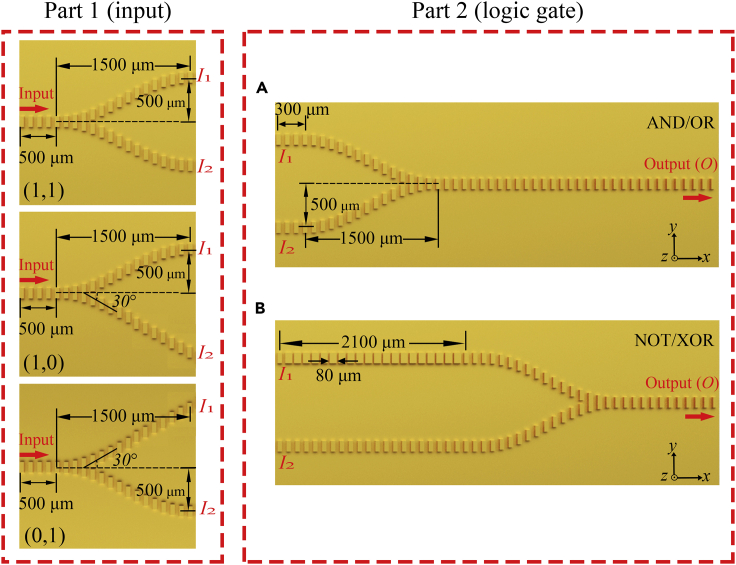
Sketch of Two-Input-Port Logic Gates in Top View Part 1 provides different input patterns for the logic gates. Part 2 performs logical calculation of AND/OR gates (A) and NOT/XOR gates (B).

The proposed two-port logic gates with detailed structural parameters are provided in Figure 2. The structure is divided into two parts. Part 1 provides input signals for the logic gates and Part 2 performs the logical operation. In the device, the two ports denoted by *I*_1_ and *I*_2_ outputs from Part 1 are taken as channels for the input signals of the logic gate. Since the total input intensity is fixed, different structures are used to realize the four input situations, while ensuring that the intensity of logic 1 in different states remains unchanged. For the input (1, 1), the input intensity is divided into two paths by a beam splitter and connected to the logic gates through S-bend waveguides based on the cosine function. For the (0, 1) and (1, 0) input singles, the input intensity is also divided into two paths, one of which is connected to the logic gate as logic 1, and the other is not connected to the logic gate as logic 0. The input signal of logic 0 is realized by guiding the SPPs to the outside of the structure with an inclined straight waveguide. This design is to realize the presence or absence of signals in the integration of actual logic gates. As explained earlier, such a beam-splitting design is not necessary but is used to obtain more easily two or more signal sources with specific amplitude or initial phase in the experiment. In the practical application of the proposed logic gate, only the second part is connected to the system. For logic gates in Part 2, the phases of the two arms are controlled by changing the length of the metallic pillars. Destructive interference can be reached in the output waveguide when the phase difference of the two arms is an odd integer multiple of *π*, whereas constructive interference can be reached when the phase difference is an even integer multiple of *π*. The remainder port denoted by *O* is used as the output of the logic gate.

**Figure 3:**
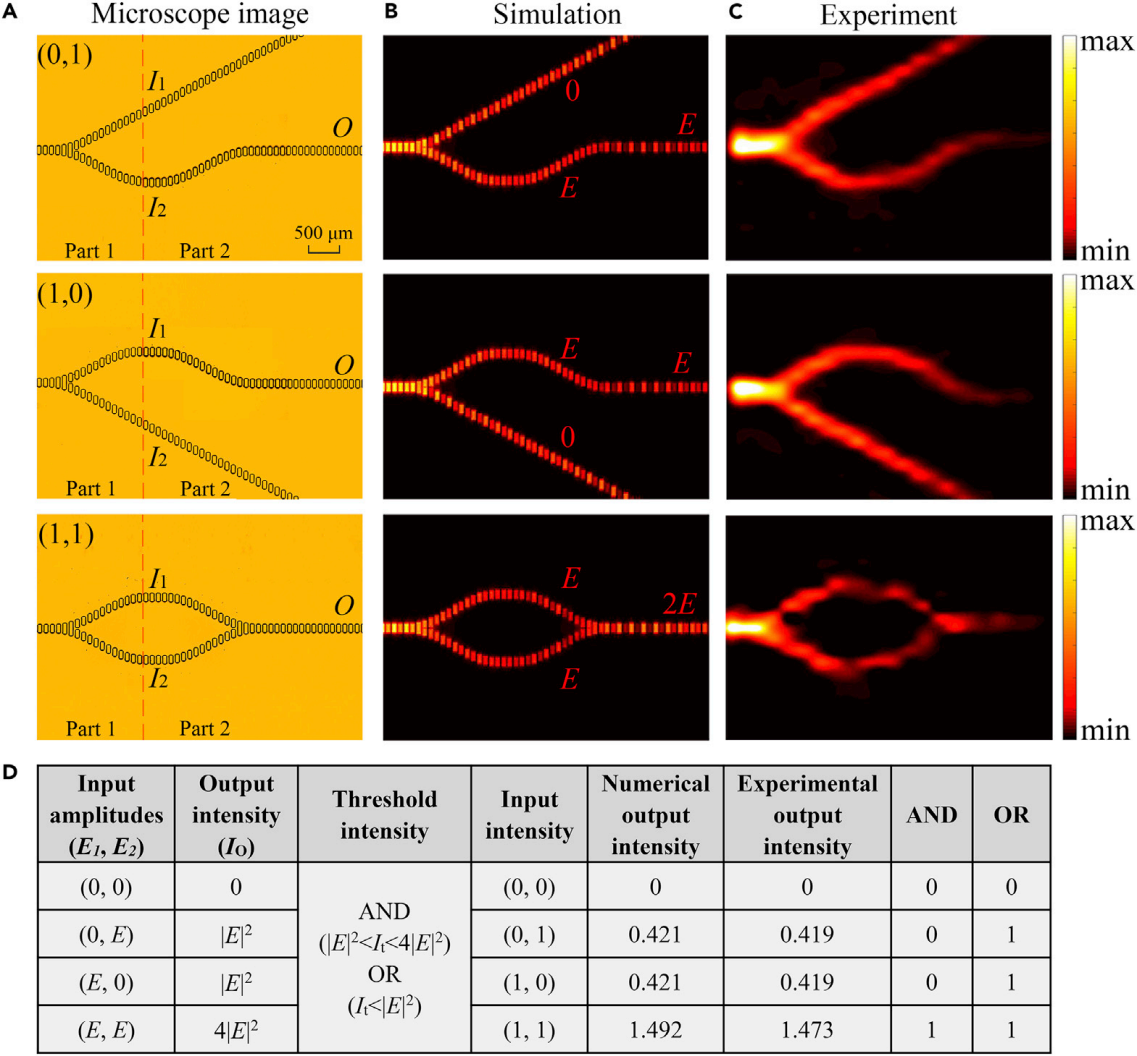
Performance of AND/OR Gates with Different Input Signals (A) Optical microscopy photos of fabricated AND/OR gates in top view. Part 1 provides different input patterns for the logic gates and Part 2 performs logical calculation of the AND/OR gates. Note that an inclined straight waveguide is used to realize a logic 0 input. (B) Simulated results for the normalized power |*E*_*z*_|^2^ distributions corresponding to (0, *E*), (*E*, 0), and (*E*, *E*) inputs in a horizontal plane slightly above (at 100 μm) the surface of each structure at 0.58 THz. (C) Experimental results for the normalized power |*E*_*z*_|^2^ distributions corresponding to (0, *E*), (*E*, 0), and (*E*, *E*) inputs in a horizontal plane slightly above (at 100 μm) the surface of each structure at 0.58 THz. (D) Truth table of the logic AND/OR gates. Numerical and experimental outputs are normalized with respect to the intensity of *I*_*1*_ or *I*_*2*_ outputs from Part 1, namely, the value of the (1, 0) input.

Figure 3A shows the optical microscopy photos of the fabricated AND/OR gates realized by constructive interference. For the AND/OR gates displayed in Figure 3A, the surface wave propagates along the waveguide and divides into the two straight waveguides with the same propagation vector *k*_1_ after Part 1 and finally combines by the Y-shaped waveguide to form the output. The distances from the input ports of the two waveguides to the interaction point have exactly the same value, which will ensure constructive interference in the output waveguide.

The input and output of the logic gate are all encoded by their intensities. Two signals are transmitted to the junction from the input ports and then reach the output port. The amplitudes of the two transmitted signals from *I*_1_ and *I*_2_ are *E*_1_ and *E*_2_, respectively. The constructive or destructive interference between *E*_1_ and *E*_2_ is determined by the phase difference of the input signals, resulting in a different output *E*_o_. The decision mechanism of the logic gates is based on binary on-off-keying, that is, the amplitude of the signal is used as the criterion of the logic (Li et al., 2016). For the inputs, “on” and “off” are recognized to be logic 1 and logic 0, respectively. To facilitate observation and measurement, the threshold intensity *I*_t_ = |*E*_t_|^2^ is defined to determine the Boolean value of the output. If the output intensity *I*_o_ is larger than the threshold *I*_t_, the output value is logic 1; otherwise, it is logic 0. By correctly defining the values of the threshold intensity, the two-port device can achieve the logic operations of AND, OR, NOT, and XOR gates, respectively (Fu et al., 2012; Birr et al., 2015).

To verify the functions of the logic gates, simulations based on the time domain solver of CST Microwave Studio are performed. As the source, the SPPs are excited by a plane wave irradiated vertically on the excitation region of the sample from the bottom side. In order to obtain a maximized and optimized SPP field, we calculated the transmission spectra of the logic gates in the paper. The results show that the center frequency excited and supported by the structure is 0.58 THz. In order to obtain the maximum output energy, the devices are designed at an operating frequency of 0.58 THz. Figure 3B shows the simulated normalized power |*E*_z_|^2^ distributions for different input signals of the AND/OR gates with a scanning area of 6.5 × 3 mm^2^ at 0.58 THz. Clearly, the designed logic gates show high performance. Figure 3C shows the corresponding measured images, which are in good agreement with the simulations. For the experimental demonstration, waveguide structures are fabricated on a 4-inch silicon wafer by optical lithography and deep reactive ion etching techniques. Then, the chip is metallized with a 200-nm-thick gold film in a gold sputter coater. The thickness of gold is selected based on the penetration depth of the THz wave in the metal. The fabricated structures are then experimentally characterized using a fiber-optic scanning near-field THz microscopy system, as described in detail elsewhere (Yuan et al., 2019, 2020). The truth table of the AND/OR gates is given in Figure 3D. In the structure of the two-input logic gates, the surface wave excited by the grating is divided into two beams of equal energy after passing through Part 1, so *E*_1_ and *E*_2_ are set to be *E*. The output intensity *I*_o_ is 0 for input (0, 0), |*E*|^2^ for inputs (0, *E*) and (*E*, 0), and 4|*E*|^2^ for input (*E*, *E*). When *I*_t_ is chosen to be below |*E*|^2^, the output state is logic 1 for input (0, *E*), (*E*, 0), and (*E*, *E*), realizing the OR logic function. When *I*_t_ is chosen to be above |*E*|^2^ and below 4|*E*|^2^, the output is logic 1 only for (*E*, *E*) input, corresponding to the AND logic operation. The normalized output power for different situations are obtained and presented in Figure 3D. For each situation, the measured output power is obtained by integrating the power |*E*_z_|^2^ near the same position of the output port of the waveguides. The results are normalized by the input power measured in the initial position of the interferometer arm to eliminate the influence of propagation loss. As can be seen, the two logic operations can be implemented by correctly defining the threshold intensity. The experimental intensity contrast is about 5.46 dB for the AND gate. The experimentally measured total loss is about 3.78 dB for the AND/OR gates. The loss is calculated as the ratio of the power coupled to the fundamental mode of the input waveguide to the output power: Loss = 10log(*P*_in_/*P*_out_). Since in our analysis the actual metallic loss and radiative loss inside the waveguides are the only source of loss. The insertion loss of the AND/OR gates is about 1.87 dB, which is extracted by comparing the total loss of the structure with that of a straight waveguide of the same length. The corresponding propagation loss of the straight waveguide is about 1.06 dB/mm.

**Figure 4:**
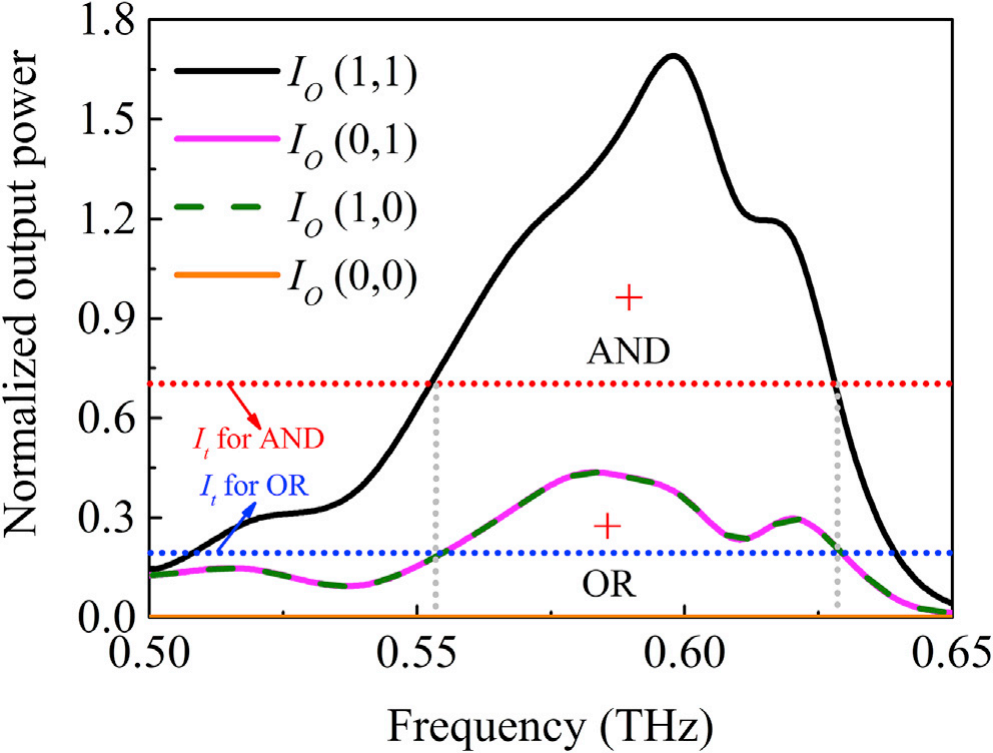
Normalized Output Power at Port *O* as a Function of Frequency for AND/OR Gates

The normalized output power at port *O*, *I*_o_ (*I*_1_, *I*_2_), as a function of frequency is shown in Figure 4. The output powers for single inputs *I*_o_ (0, 1) and *I*_o_ (1, 0) are shown as the solid pink line and dotted green line, respectively. The value of the power is obtained by integrating the longitudinal component of the Poynting vector on vertical planes near the input and output of the waveguide. The integrating regions have the same dimension of 600 μm × 600 μm to ensure the correct calculation of energy distribution. The power is then normalized by that of *I*_1_ or *I*_2_ output from Part 1. The high output power of *I*_o_ (1, 1) due to complete interference is attributed to the single-mode characteristics and structural symmetry of the waveguide, as shown by the black line. By defining the corresponding threshold intensity *I*_t_, the logic functions can be realized. For a threshold value of *I*_t_ = 0.7*I*_1_, the AND operation can be realized. For a threshold value of *I*_t_ = 0.2*I*_1_, the device functions as an OR gate (Pan et al., 2013). The dotted red and blue lines in Figure 4 show the operation bandwidths of the AND and OR logic gates at this threshold, respectively, which is about 70 GHz. Defined as the highest power ratio of logic 1 to logic 0 at the operation wavelength, the intensity contrast of a logic gate is a factor that determines the suitability of the design. The intensity contrast for the AND gate is about 5.49 dB. As shown by the red cross marks in the figure, the threshold intensity can be selected flexibly. For a given threshold strength, these logic operations can be implemented in a wide range, which makes the device have a large tolerance.

**Figure 5:**
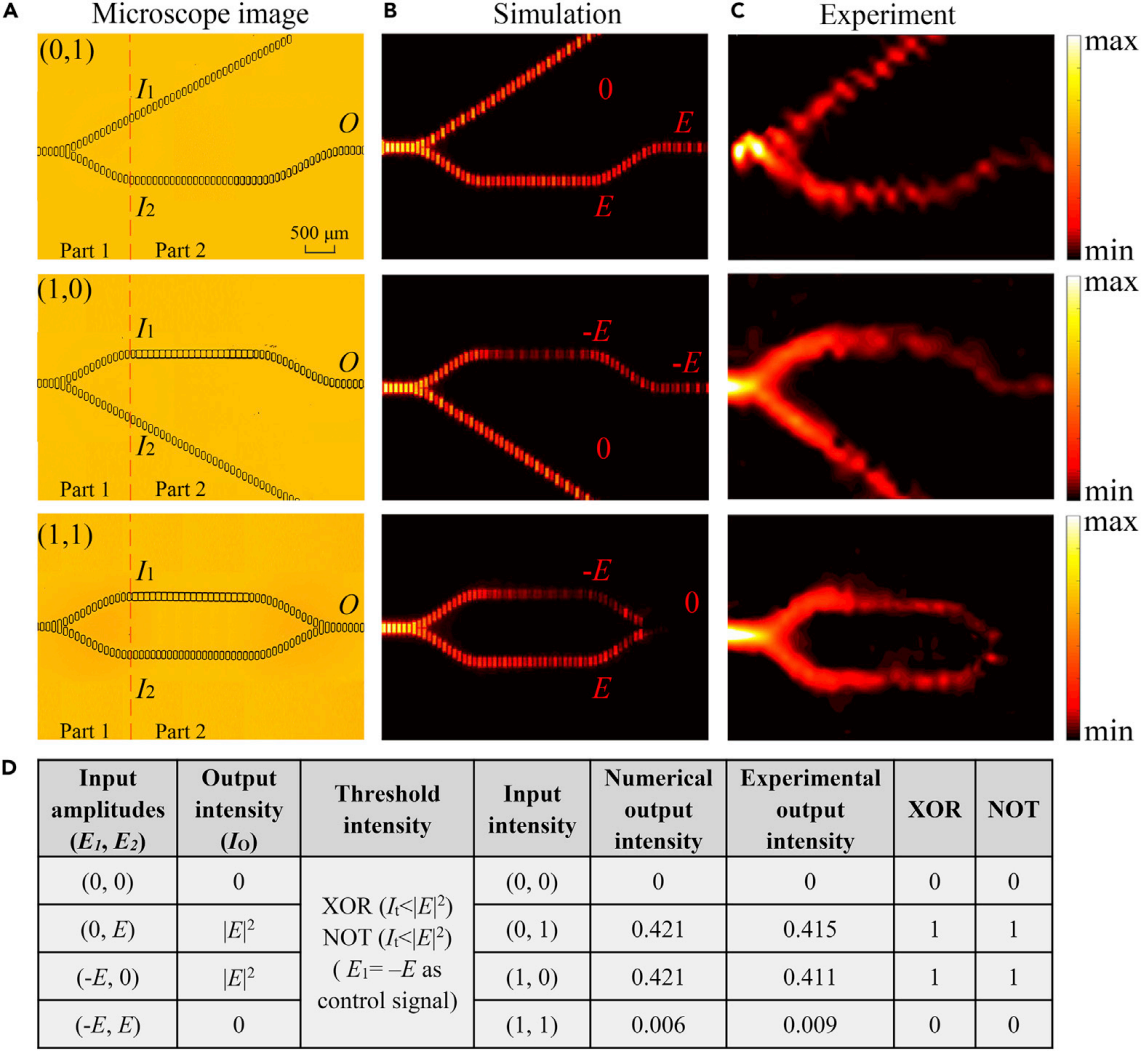
Performance of NOT/XOR Gates with Different Input Signals (A) Optical microscopy photos of fabricated NOT/XOR gates in top view. (B) Simulated results for the normalized power |*E*_*z*_|^2^ distributions corresponding to (0, *E*), (*E*, 0), and (*E*, *E*) inputs in a horizontal plane slightly above (at 100 μm) the surface of each structure at 0.58 THz. (C) Experimental results for the normalized power |*E*_*z*_|^2^ distributions corresponding to (0, *E*), (*E*, 0), and (*E*, *E*) inputs in a horizontal plane slightly above (at 100 μm) the surface of each structure at 0.58 THz. (D) Truth table of the logic NOT/XOR gates.

Figure 5A shows the optical microscopy photos of the fabricated NOT/XOR gates realized by destructive interference. For our Mach-Zehnder interferometer, when the length of the unit structures of one arm changes, the mode of the SPP will change accordingly. The unit structure length of the upper arm is 80 μm, whereas that of the lower arm remains unchanged at 50 μm. At the same frequency, the wave vector of the surface wave on the upper arm changes to *k*_2_, whereas the wave vector of the lower arm is still *k*_1_. After the SPPs have propagated a certain distance *L*, the difference between these two wave vectors at the same frequency will lead to a phase difference. When the accumulated phase difference satisfies Equation 1:

(Equation 1) $$\left(k_{2}-k_{1}\right)L=\left(2n+1\right)\pi$$

where *n* is an integer, complete destructive interference between the two waves will occur. When *n* = 0, for the two arms with a unit length of 50 and 80 μm, respectively, the propagation length *L* = *π*/(*k*_2_-*k*_1_) required to accumulate a phase difference of *π* is 2,100 μm at 0.58 THz.

For destructive interference, the complex amplitudes of the outputs for the individual inputs *E*_1_ and *E*_2_ are –*E* and *E*, respectively. Because of the symmetry of the gate structure, complete destructive interference can be realized at port *O*, resulting in large interference visibility. Figure 5B shows the simulated normalized power |*E*_z_|^2^ distributions for different input signals of the NOT/XOR gates with a scanning area of 8 × 3 mm^2^ at 0.58 THz. Figure 5C shows the corresponding measured images, which are in good agreement with the simulations.

The truth table of the NOT/XOR gates is shown in Figure 5D. The output intensity *I*_o_ is 0 for inputs (0, 0) and (–*E*, *E*) and |*E*|^2^ for inputs (0, *E*) and (–*E*, 0). When *I*_t_ is chosen to be below |*E|*^2^, the output state is logic 1 for inputs (0, *E*) and (–*E*, 0), realizing the XOR logic function. If the signal –*E* at port *I*_1_ is used as the control signal, for the inputs 0 and –*E* at port *I*_2_, i.e., (–*E*, 0) and (–*E*, *E*), the output states are just opposite to the input states, which leads to the function of a NOT gate. The experimental intensity contrast is about 16.60 dB for the XOR and NOT gates. The experimentally measured total loss is about 3.82 dB for the NOT/XOR gates, and the insertion loss is about 1.91 dB.

**Figure 6:**
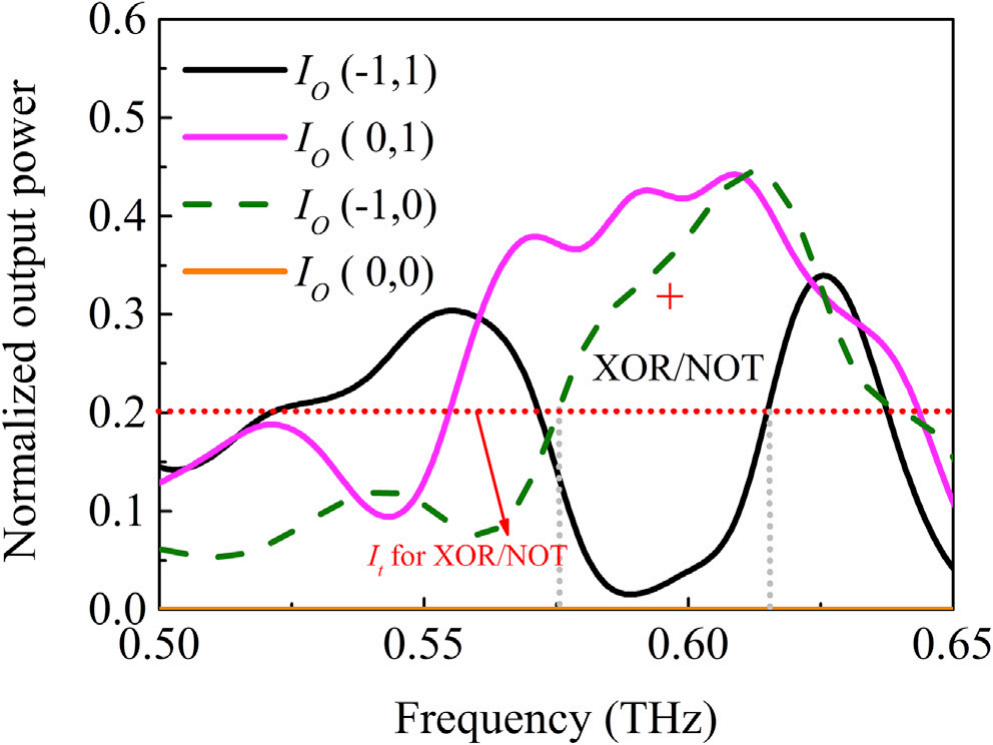
Normalized Output Power at Port *O* as a Function of Frequency for NOT/XOR Gates

The normalized output power at port *O*, *I*_o_ (*I*_1_, *I*_2_), as a function of frequency is shown in Figure 6. The output powers for the inputs *I*_o_ (0, 1) and *I*_o_ (1, 0) as indicated by the solid pink line and dotted green line, respectively, remains unchanged, whereas the output for *I*_o_ (−1, 1) decreases sharply owing to the destructive interference. It should be noted that there is a slight difference in the amplitudes of the transmitted powers owing to the structural difference between the two arms, so complete destructive interference cannot be fully realized. For a threshold value of *I*_t_ = 0.2*I*_1_, the XOR and NOT operations can be realized. The dotted red line in Figure 6 shows the operation bandwidth of the logic gates at this threshold, which is about 55 GHz. The intensity contrast is high, about 18.46 dB for the XOR and NOT gates.

### Logic Gates with Three Input Ports

**Figure 7:**
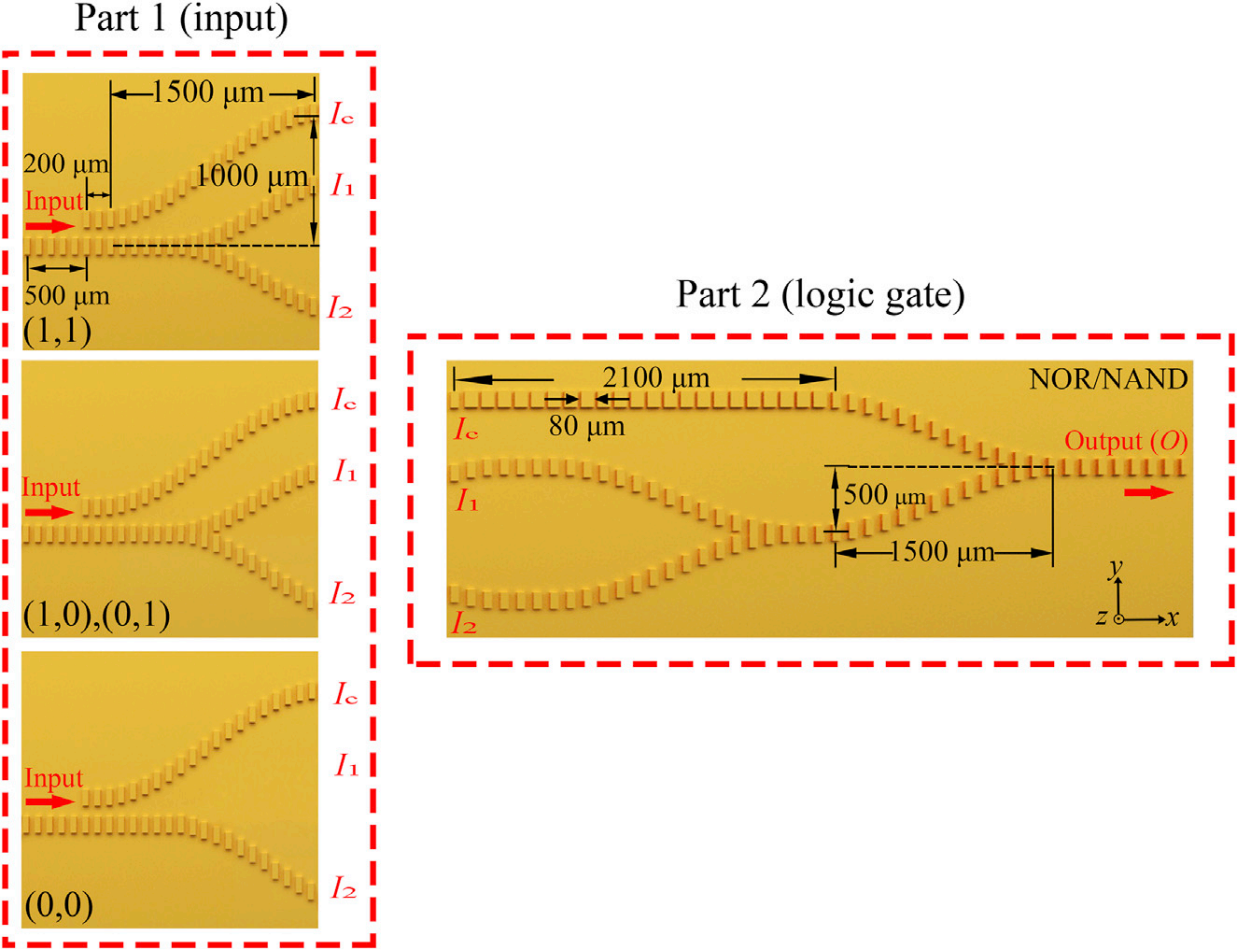
Sketch of Three-Input-Port Logic Gates in Top View Part 1 provides different input patterns for the logic gates. Part 2 performs logical calculation of NOR/NAND gates.

The NOR and NAND logic gates are composed of two sets of spoof SPP waveguides in a Mach-Zehnder interferometer configuration, as shown in Figure 7. These logic functions are composite operations of the functions described in the previous section, so they can be achieved by cascading a NOT gate with an OR and AND gate, respectively (Fu et al., 2012). Similarly, each structure is divided into two parts, where Part 1 provides input signals for the logic gates and Part 2 performs the logic calculations. The logic calculation section includes two-level operations. The first-level operations are the same as the logic gates with two input ports described above. The AND/OR and NOT/XOR gates based on the input signals *I*_1_ and *I*_2_ can be first realized. To realize the 2 × 2 cascaded logic gates, a control signal *I*_C_ is added to coherently interfere with the output of the first-stage operation to determine the final output at port *O*. The value of *I*_C_ is controlled by the coupling length of the parallel waveguides in the first part. The gap between the two parallel waveguides is 80 μm and the length of the parallel section is *L*. When two identical waveguides are close to each other, there are two supermodes supported by the entire structure. The difference in the propagation constants *k*_SPP_ between these two supermodes at the same frequency will lead to a phase difference. After the SPPs have propagated a certain distance, the mode power will be shifted from one waveguide to the other in the case of identical waveguide geometries. According to the coupled-mode theory, the output powers from the two parallel waveguides can be obtained as a function of *L* (Yuan et al., 2020). Here, the section lengths *L* are calculated to be 200 μm for the NOR/NAND gates.

**Figure 8:**
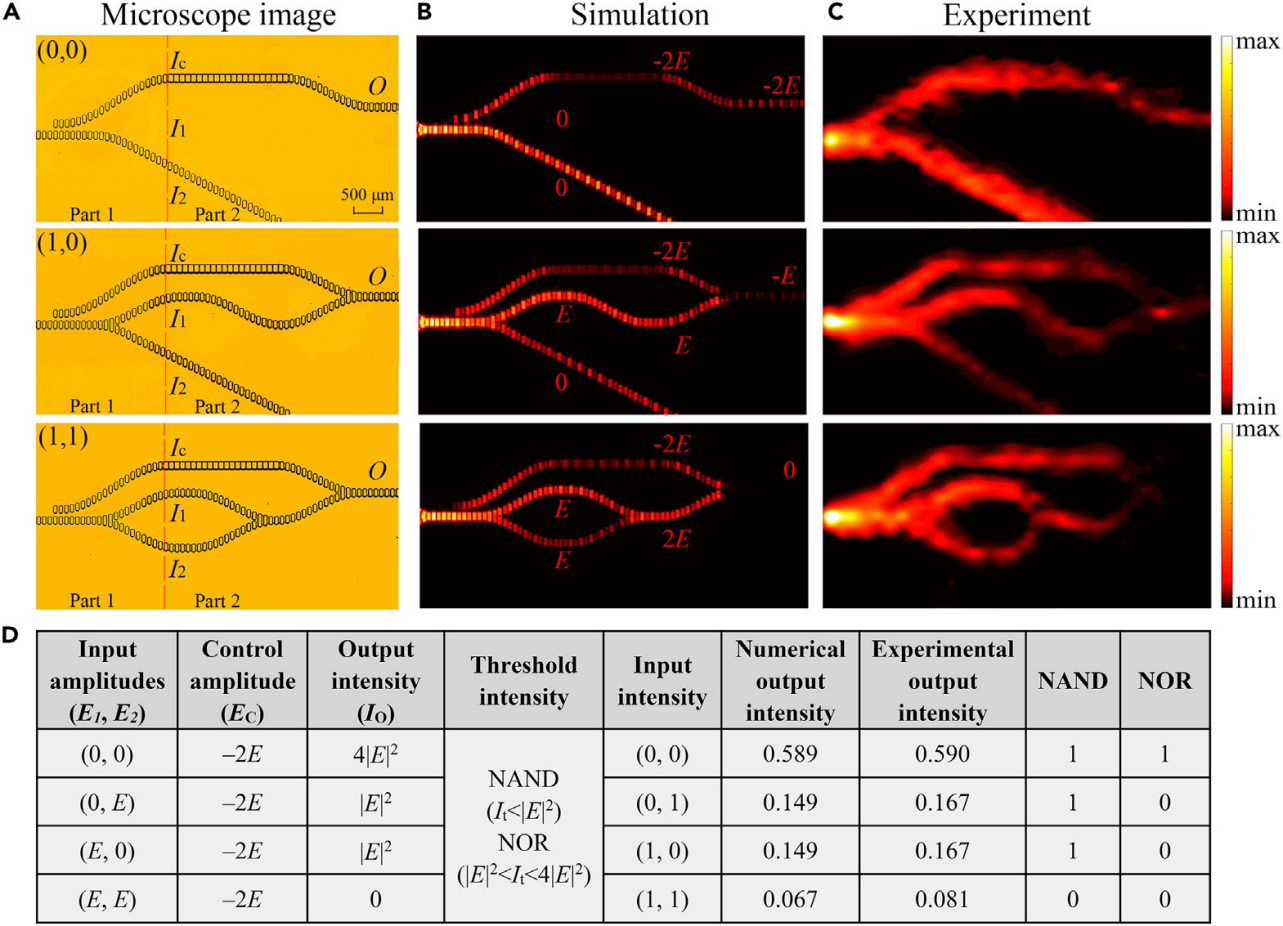
Performance of NOR/NAND Gates with Different Input Signals (A) Optical microscopy photos of fabricated NOT/XOR gates in top view. (B) Simulated results for the normalized power |*E*_*z*_|^2^ distributions corresponding to (0, *E*), (*E*, 0), and (*E*, *E*) inputs in a horizontal plane slightly above (at 100 μm) the surface of each structure at 0.58 THz. (C) Experimental results for the normalized power |*E*_*z*_|^2^ distributions corresponding to (0, *E*), (*E*, 0), and (*E*, *E*) inputs in a horizontal plane slightly above (at 100 μm) the surface of each structure at 0.58 THz. (D) Truth table of the logic NOT/XOR gates.

Figure 8A shows the optical microscopy photos of the fabricated NOR/NAND gates. Figure 8B shows the simulated normalized power |*E*_z_|^2^ distributions for different input signals of the NOR/NAND gates with a scanning area of 8 × 3 mm^2^ at 0.58 THz. Figure 8C shows the corresponding measured images. Again, good agreement between the two is obtained. Because of the actual metallic loss and radiative loss inside the waveguides, the SPP intensities at the end of the waveguides are lower compared with the simulated gate structures. Notwithstanding the inevitable loss, the structures still perform remarkably well without affecting the function of the logic gates.

The truth table of the NOR/NAND gates is shown in Figure 8D. For the first gate, to perform the AND/OR logic operations, the two input signals from ports *I*_1_ and *I*_2_ are in phase (Figures 3A and 3D). An out-of-phase control signal from port *I*_C_ can invert the output of the first gate as a NOT gate. As shown in Figure 8D, when *E*_*C*_ is twice as large as *E*_*1*_ and *E*_*2*_ (*E*_*1*_ = *E*_*2*_ = *E*) with a phase difference of *π*, the output intensity *I*_*o*_ is 0 for input (*E*, *E*), |*E*|^2^ for inputs (0, *E*) and (*E*, 0), and 4|*E*|^2^ for input (0, 0). When the threshold value *I*_t_ is chosen between |*E*|^2^ and 4|*E*|^2^, the output state is logic 1 for input (0, 0) and the NOR operation can be realized. For a threshold value *I*_t_ below |*E*|^2^, the output state is logic 1 for inputs (0, 0), (0, *E*) and (*E*, 0), and the function of a NAND gate can be realized. The experimental intensity contrast is about 5.48 dB for the NOR gate and 3.14 dB for the NAND gate. The experimentally measured total loss is about 7.75 dB for the NOR/NAND gates, and the insertion loss is about 3.72 dB.

**Figure 9:**
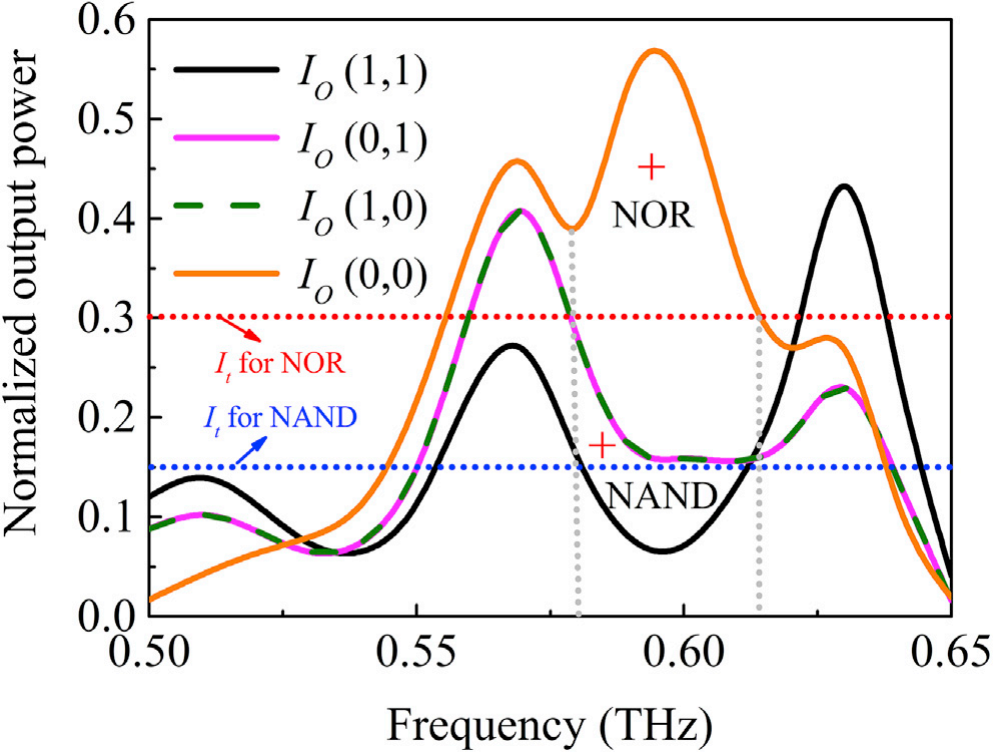
Normalized Output Power at Port *O* as a Function of Frequency for NOR/NAND Gates

Figure 9 shows the variation of the output power at port *O* as a function of frequency for different inputs. The red crosses mark the corresponding area and power threshold for the three logic functions. For |*E*|^2^ < *I*_t_ < 4|*E*|^2^, the NOR operation can be realized. For *I*_t_ < |*E*|^2^, the device functions as a NAND gate. For a threshold value of *I*_t_ = 0.3*I*_1_, the NOR operation can be realized. For a threshold value of *I*_t_ = 0.15*I*_1_, the device functions as a NAND gate. The dotted red and blue lines in Figure 9 show the operation bandwidths of the NOR and NAND logic gates at this threshold, respectively, which is about 50 GHz. The intensity contrast for the output values of logic 1 and logic 0 is about 5.97 dB for the NOR gate and 3.47 dB for the NAND gate.

## Discussion

Since the coupling between waveguides is the main limitation to be considered to minimize the device dimensions, according to the coupled mode theory, the coupling between the waveguides decreases as the distance between the waveguides increases and reduces almost to zero when the distance is about 280 μm (Yuan et al., 2020). At this point, it can be assumed that there is no coupling between the two waveguides, and this distance between them is defined as the minimum routing distance.

The operation speed of the logic gates proposed in this paper is mainly determined by the time required for the surface waves to propagate on the waveguide. Here, the propagation length of the logic gates is defined as the output position of the beam splitter to the interference position of the logic gates. For the AND/OR gates, the required operation time is about 15 ps, and for the NOT, XOR, NAND, and NOR gates, the operation time is about 35 ps.

In summary, a series of novel THz basic logic operations based on spoof SPP waveguides are designed and experimentally demonstrated based on the linear interference effect. A single Mach-Zehnder interferometer can function as an AND, OR, NOT, or XOR logic gate. By using two cascaded Mach-Zehnder interferometers with one arm as the control input, NAND and NOR operations can be accomplished. The SPP waveguide systems have been fabricated on an area of 10 × 5 mm^2^ by high precision lithography, while integrating several cascaded SPP waveguides and logic elements on the same substrate. Assembles of these gates can lead to complex functionalities and open up an avenue toward developing THz plasmonic computing chips.

### Limitations of the Study

Experiments to investigate XNOR gate as well as assembles of the gates were not included in this work and require further investigation.

### Resource Availability

#### Lead Contact

Further information and requests for resources should be directed to and will be fulfilled by the Lead Contact, Associate Professor Yanfeng Li (yanfengli@tju.edu.cn).

#### Materials Availability

This study did not generate new unique reagents.

#### Data and Code Availability

This study did not generate/analyze datasets/code.

## Methods

All methods can be found in the accompanying Transparent Methods supplemental file.

## Supplemental Information

Document S1. Transparent Methods and Figures S1–S3
