# Supplementary material for: Terahertz Spoof Surface Plasmonic Logic Gates
Source: iScience. 2020 Oct 15;23(11):101685. doi: 10.1016/j.isci.2020.101685 (PMC7607436; doi:10.1016/j.isci.2020.101685)
Supplement: Document S1. Transparent Methods and Figures S1–S3 [file mmc1.pdf]

iScience, Volume *23*

Supplemental Information

Terahertz Spoof Surface

Plasmonic Logic Gates

Mingrui Yuan, Qingwei Wang, Yanfeng Li, Yuehong Xu, Quan Xu, Xueqian Zhang, Xixiang Zhang, Jiaguang Han, and Weili Zhang

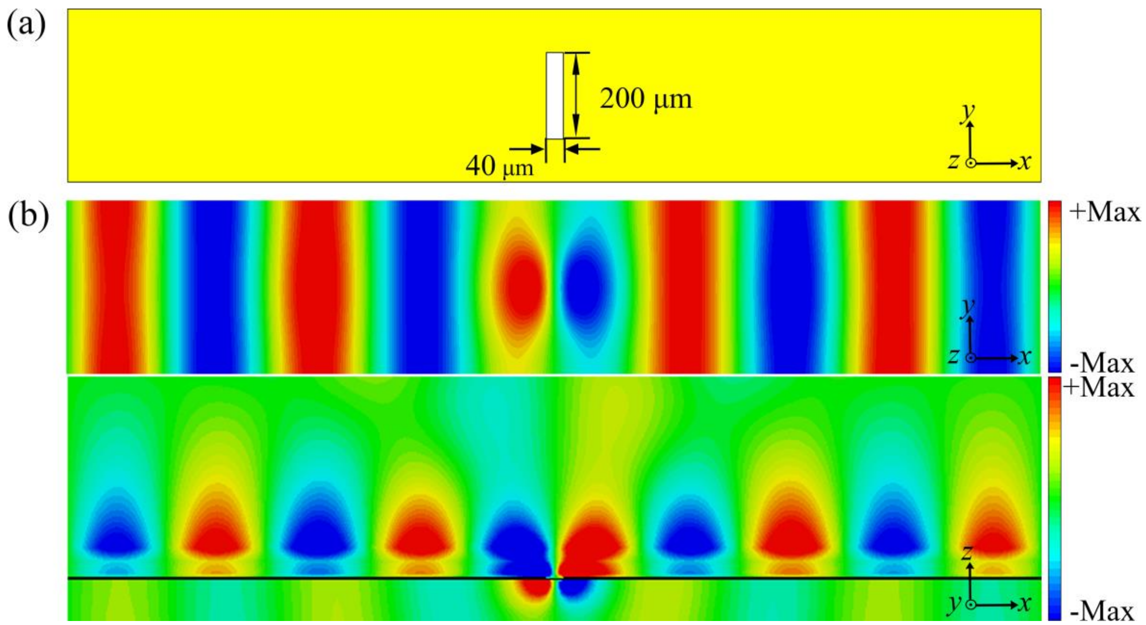

Figure S1. Performance of the excitation region. (a) Sketch of the metallic slit. (b) Normalized electric component (E_z) distributions in the *xy* (upper) and *xz* (lower) cross-sections excited by the metallic slit resonator at 0.58 THz. Related to **Figure 1**.

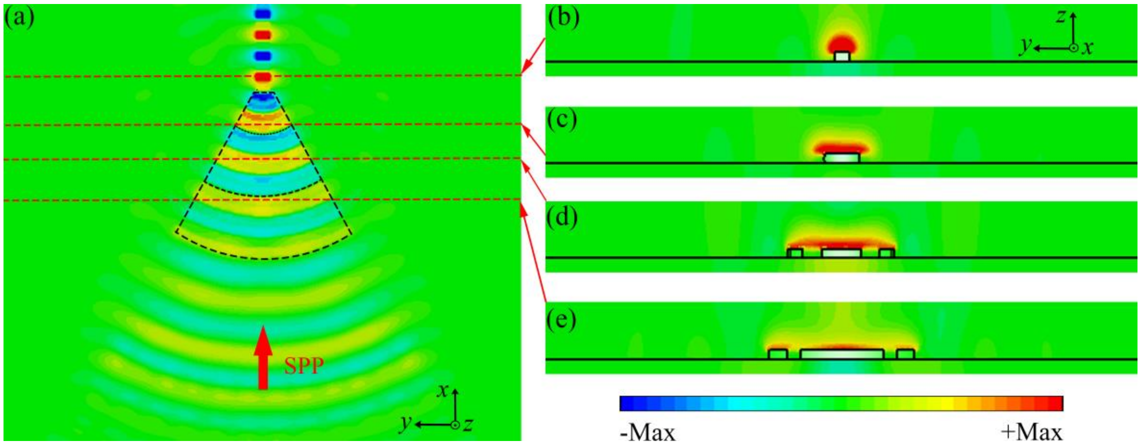

Figure S2. Performance of the coupling region. (a) Normalized electric component (E_z) distribution in a horizontal plane slightly above (100 μm) the height of the annular sector-shaped hole array excitation region as well as the annular sector-shaped column array coupling region (top view). (b)-(e) Normalized electric component (E_z) distributions in transverse vertical planes (longitudinal views) at selected locations shown by red dashed lines in (a). Related to **Figure 1**.

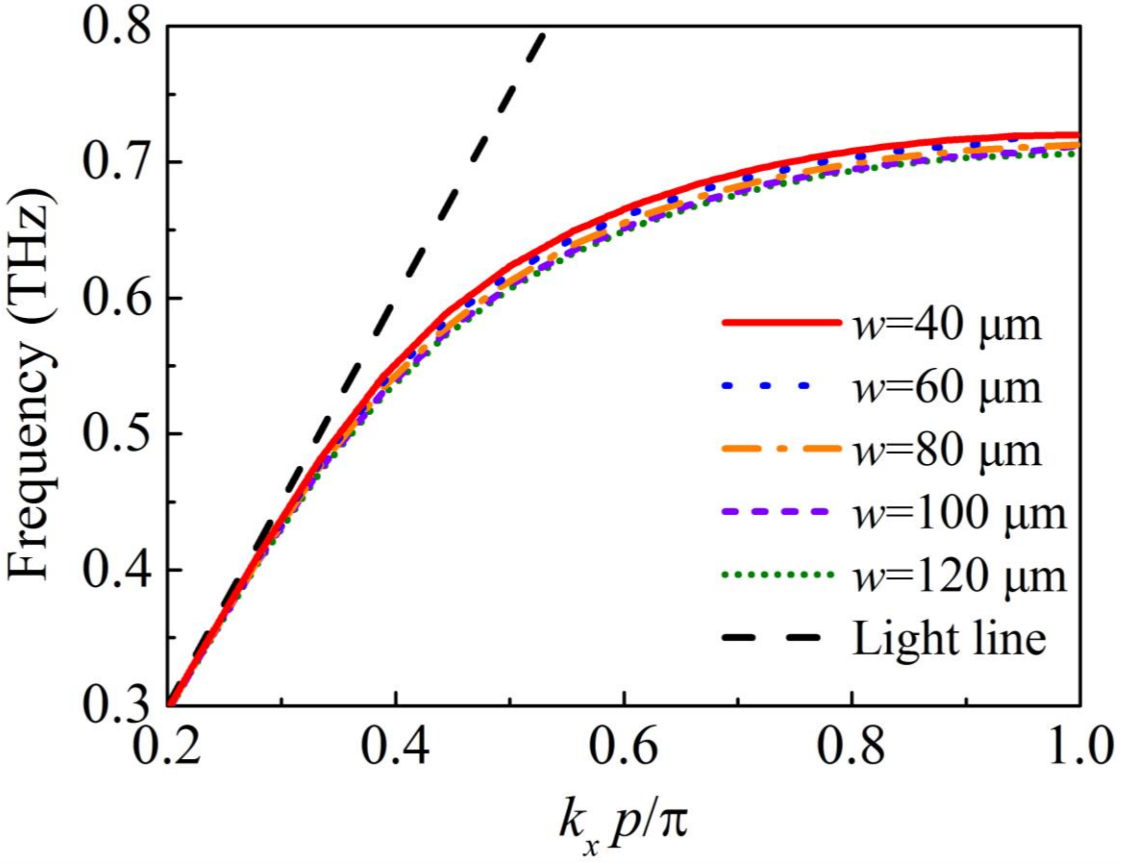

Figure S3. Dispersion relation of SPP mode for one row of metallic pillars with different widths from 40 μm to 120 μm. Related to **Figure 1**.

Transparent Methods

Design of the excitation region

Due to the lack of direct SPP sources in the THz regime, we need to couple free-space THz waves into SPPs. Similar to the optical regime, the key to excite THz SPPs is based on the momentum match (Maier, 2007). The method to couple the free-space THz wave to SPPs is the grating approach, which provides additional reciprocal lattice vectors $G = 2m\pi/p$, where p is the period of the grating and m is an integer (Zhang et al., 2020). When the THz wave interacts with the grating, the superposition of the tangential wave vector of the THz wave $k_0\sin\beta$ and the reciprocal lattice vectors of the grating G results in new wave vectors. At a certain value of m, the corresponding new wave vector can match that of the THz SPPs at a certain frequency, at which the THz SPPs can be excited. In this work, a metasurface grating consisting of metallic slits are applied to excite the THz SPPs on a waveguide made of metallic pillar structures (Ebbesen et al., 1998; Yin et al., 2005). To effectively convert free-space THz waves to SPPs, the lateral size of the grating is designed to match the incident THz beam size. Besides, the holes are curved to match the structure of the excitation region to obtain a higher coupling efficiency.

The metacoupler is composed of metallic slit resonators, as shown in Fig. S1 (a). The normalized electric distributions (E_z) of an metallic slit resonator in the xy (upper) and xz (lower) cross-sections at 0.58 THz are displayed in Fig. S1 (b). Periodic boundaries are applied in both the x and y directions, and open boundaries are applied in the z direction. It can be seen that most of the electric field exists as the E_z component and ise confined to propagate at the interface.

Design of the coupling region

To fully utilize the excited SPPs, an arc-shaped metasurface composed of the same metallic columns are designed to guide the excited SPPs to the waveguide. Due to the poor localization of the metal surface to

the SPP field in the THz regime, the SPP wave propagates forward in the form of half space wave (Zhang et al., 2020). Therefore, the SPP field will be easily coupled to the waveguide with a small loss. When the SPP field is coupled to the funnel-shaped metasurface, since the effective index of the SPP mode is rather insensitive to the lateral width of the pillar, a compact taper is able to laterally compress the mode size down (Martin-Cano et al., 2010). Figure S2(a) shows the normalized electric component (E_z) distribution in a horizontal plane slightly above (100 μm) the height of the annular sector-shaped hole array excitation region as well as the annular sector-shaped column array coupling region, showing vividly the process of SPP field excitation and coupling. The area within the black dotted lines is the annular sector-shaped column array coupling region. Panels (b)-(e) are cross-section field distributions at different positions along the column array, showing how the SPP mode size is gradually reduced by the tapered metasurface design. Remarkably, reflection is small and some incoming power is lost as radiation loss.

Figure S3 shows the dispersion relation for the fundamental SPP mode of the waveguide. The width of the metallic pillar *w* is varied from 40 μm to 120 μm to investigate its effect on the surface wave propagation. The important point to note is that although the cutoff frequency becomes smaller when the width is increased from 40 μm to 120 μm, the dispersion relation of the SPP mode is generally insensitive to the width (Martin-Cano et al., 2010). This explains why the funnel-shaped metasurface composed of tapered metallic pillars can couple the SPP wave efficiently into the waveguide as shown in Fig. S3.

Supplemental references

Ebbesen, T., Lezec, H., Ghaemi, H., Thio, T. and Wolff, P., (1998). Extraordinary optical transmission through sub-wavelength hole arrays. Nature, *391*, 667.

Martin-Cano, D., Nesterov, M., Fernandez-Dominguez, A., Garcia-Vidal, F., Martin-Moreno, L., and Moreno, E. (2010). Domino plasmons for subwavelength terahertz circuitry. Optics Express *18*, 754.

Maier, S. (2007). *Plasmonics: Fundamentals and Applications* (Springer US).

Yin, L., Vlasko-Vlasov, V., Pearson, J., Hiller, J., Hua, J., Welp, U., Brown, D. and Kimball, C., (2005). Subwavelength focusing and guiding of surface plasmons. Nano Letters, *5*, 1399.

Zhang, X., Xu, Q., Xia, L., Li, Y., Gu, J., Tian, Z., Ouyang, C., Han, J., and Zhang, W. (2020). Terahertz surface plasmonic waves: a review. Advanced Photonics *2*, 014001.
